# Discovery and characterization of the feline miRNAome

**DOI:** 10.1038/s41598-017-10164-w

**Published:** 2017-08-23

**Authors:** Alessandro Laganà, Wessel P. Dirksen, Wachiraphan Supsavhad, Ayse Selen Yilmaz, Hatice G. Ozer, James D. Feller, Kiersten A. Vala, Carlo M. Croce, Thomas J. Rosol

**Affiliations:** 1 Department of Molecular Virology, Immunology and Medical Genetics, Comprehensive Cancer Center, The Ohio State University, Columbus, OH USA; 2 Department of Veterinary Biosciences, College of Veterinary Medicine, The Ohio State University, Columbus, OH USA; 3 Department of Biomedical Informatics, Comprehensive Cancer Center, The Ohio State University Wexner Medical Center, Columbus, OH USA; 4 Department of Genetics and Genomic Sciences, Icahn School of Medicine at Mount Sinai, New York, NY USA; 5 Department of Pathology, Faculty of Veterinary Medicine, Kasetsart University, Bangkok, Thailand

## Abstract

The domestic cat is an important human companion animal that can also serve as a relevant model for ~250 genetic diseases, many metabolic and degenerative conditions, and forms of cancer that are analogous to human disorders. MicroRNAs (miRNAs) play a crucial role in many biological processes and their dysregulation has a significant impact on important cellular pathways and is linked to a variety of diseases. While many species already have a well-defined and characterized miRNAome, miRNAs have not been carefully studied in cats. As a result, there are no feline miRNAs present in the reference miRNA databases, diminishing the usefulness of medical research on spontaneous disease in cats for applicability to both feline and human disease. This study was undertaken to define and characterize the cat miRNAome in normal feline tissues. High-throughput sequencing was performed on 12 different normal cat tissues. 271 candidate feline miRNA precursors, encoding a total of 475 mature sequences, were identified, including several novel cat-specific miRNAs. Several analyses were performed to characterize the discovered miRNAs, including tissue distribution of the precursors and mature sequences, genomic distribution of miRNA genes and identification of clusters, and isomiR characterization. Many of the miRNAs were regulated in a tissue/organ-specific manner.

## Introduction

MicroRNAs (miRNAs) are small non-coding RNAs that act as post-transcriptional regulators of gene expression. Their mature form (≅18–22 nt long) is incorporated into a protein complex called RISC (RNA-induced silencing complex) to which they confer binding specificity to target mRNA molecules^1, 2^. miRNAs can bind their targets through partial or perfect complementarity and inhibit their translation or promote their degradation. Target recognition is often mediated by the seed region, a 6- to 8-nucleotide sequence at the 5′ end of the mature miRNA that forms Watson–Crick base pairs with the cognate target, although a supplementary or compensatory role for the central and 3′ end of the miRNA has been observed^3, 4^.

miRNAs play a crucial role in many biological processes and pathways, such as apoptosis, cell cycle and proliferation, and their dysregulation is linked to a variety of diseases, including heart diseases, neurological disorders, kidney diseases and cancer^5–8^. Understanding the functions of miRNAs will provide new insights on the molecular basis of many diseases and new biomarkers for diagnosis, classification, therapy, and prognosis.

The domestic cat, *Felis catus*, is a member of the mammalian order Carnivora. In 2005, the National Human Genome Research Institute (NHGRI) endorsed a light coverage (2X) whole-genome sequencing strategy for 26 mammals, including the domestic cat^9^. In September 2011, The Genome Institute at Washington University School of Medicine, in collaboration with Agencourt Bioscience Corporation and the Broad Institute, submitted an updated *Felis catus* assembly to the NCBI for a combined genome coverage of 14X. In 2014, a genome browser was created, Genome Annotation Resource Fields — GARfield (http://garfield.dobzhanskycenter.org/), which displayed the Fca-6.2 assembly and included annotated genome features^10^. Comparative analysis of the cat genome with six mammals including humans revealed a high degree of similarity^9^.

The domestic cat serves as a model for ~250 important genetic diseases that are analogous to human disorders^9–12^. In addition, cats have metabolic and degenerative disorders and forms of cancer that mimic human conditions. This suggests that veterinary clinical research using novel drug, genetic, and interventional therapies to improve the outcome of diseased cats may also inform or translate research findings for the benefit of humans.

This study was undertaken to define the cat miRNAome and the expression and potential regulation of microRNAs (miRNAs) in normal feline tissues. Many other species have already been analyzed for miRNA expression (such as the dog), but miRNAs have not been carefully analyzed in cats. Previous work includes computational prediction of miRNAs within the cat genome^9, 10^ and miRNA high-throughput sequencing performed on a feline kidney cell line^13^. However, no high-throughput sequencing and validation has been performed on normal cat tissues, thus far. As a result, there are no feline miRNAs present in miRBase, the reference microRNA database (www.mirbase.org)^14^. In this study, high-throughput sequencing was performed on 12 different normal cat tissues. 271 candidate feline miRNA precursors, encoding a total of 475 mature sequences were identified, including several novel cat-specific miRNAs. Many of these miRNAs are regulated in a tissue-specific manner.

## Results

### Generation of RNA libraries from cat tissues and processing of sequencing data

To define and characterize the cat miRNAome, we first generated twenty-seven small-RNA libraries from different tissue samples of 9 different cats (Supplementary Table S1.1). High-throughput sequencing was performed and the raw reads were pre-processed by the SOLiD software Lifescope. Processed high-quality sequences were analyzed by miRDeep2, a computational tool to map, analyze and score deep sequencing data for the identification of known and novel miRNAs^15^ (See Materials and Methods section for extended details).

**Figure 1:**
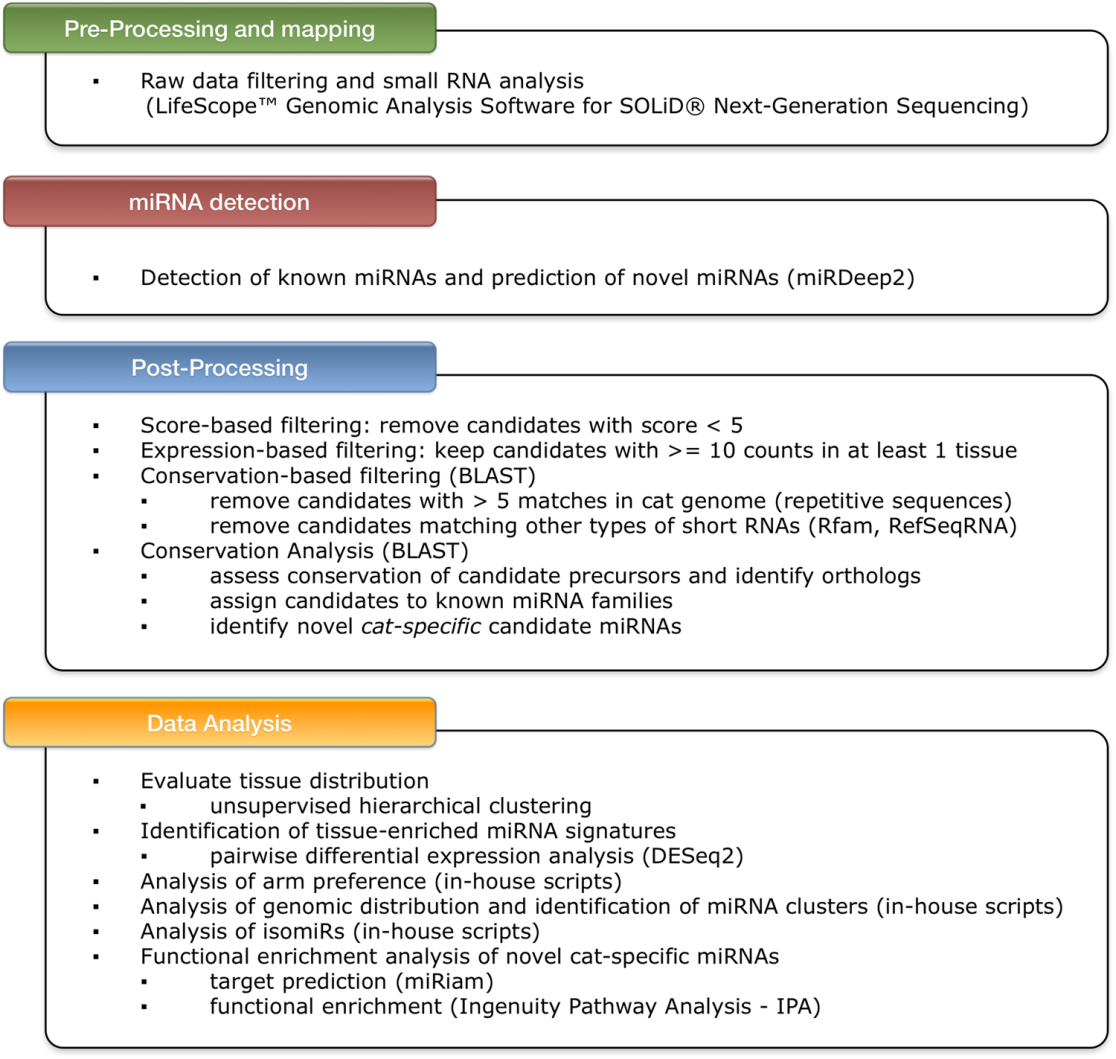
Computational pipeline of data analysis. The figure illustrates the four steps of the computational pipeline employed to analyze the RNAseq data. Pre-Processing: raw data were processed by the SOLiD software Lifescope in order to obtain good quality mappable reads. miRNA detection: this step was carried out by applying miRDeep2 to the mappable reads. Post-processing: the output of miRDeep2 was further analyzed by BLAST against different databases in order to assess conservation of the predicted miRNAs and remove sequences matching other kinds of small RNAs. Data Analysis: this step consisted of the application of a series of ad-hoc scripts for the extraction of descriptive statistics. The tool IPA was used to perform the functional enrichment analysis of potential targets of the identified miRNAs, which were predicted by the software miRiam.

MiRDeep2 analysis returned 1182 putative miRNA precursors, but only 378 of them had a score above the chosen minimum threshold of 5 and these were further processed by our in-house pipeline (see Materials and Methods; scripts are available upon request). We first removed candidates that were poorly expressed or that matched potential repetitive sequences and other types of short RNAs. We thus obtained 273 candidate miRNA precursors, encoding a total of 475 mature sequences. We analyzed conservation, tissue distribution, genomic location, arm preference and sequence variation of these novel miRNAs. The results of this analysis are summarized in Supplementary Table S1.1-S1.5 and further described in the following sections. Figure 1 illustrates the computational pipeline employed for data analysis.

### Conservation analysis

We performed a preliminary BLAST analysis against the cat genome and the Rfam and Refseq-RNA databases, in order to filter out highly repetitive sequences and sequences matching other types of short RNA. This analysis removed two candidates, as they were found to be part of longer rRNA sequences. In addition, one of these two candidates had more than five significant hits against different chromosomal areas of the cat genome (E < 2e-08).

We then performed a BLAST search for the predicted miRNA precursors across miRBase (v. 21), to assess their conservation and identify potential orthologs. For 246 out of the 271 sequences, the analysis reported a significant match with sequences from at least one other species (EValue < 1e-5), with an average of 32 orthologous miRNAs per candidate. Human, cow and dog were the species with most matches. The predicted mature sequences were highly conserved, in most cases identical to their closest homolog, and were assigned to miRBase families according to their seed sequence (see Supplementary Table S1.2 columns E, F, K and AK and Supplementary Table S1.3).

The 25 unmatched precursors were considered putative feline specific miRNAs. They encoded a total of 33 mature sequences, 28 of which had a conserved seed and were assigned to the corresponding miRBase families. It should be noted that most of these cat specific miRNAs had rather low counts and, with a few exceptions, appeared to be tissue specific. We assigned them a temporary ID based on their chromosome.

Finally, we performed an *in-silico* analysis to investigate whether there was evidence in the cat genome of the conserved miRNAs that were missed by our analysis. For this task, we considered only the 268 miRNA families that were conserved in at least five species including human. These families contained 515 different miRNA genes, 214 of which were recovered by our deep sequencing analysis (41%) (see Supplementary Table S1.2, column F). Moreover, for 16 miRNAs missed by our analysis, we found at least one other miRNA from the same family (See Supplementary Table S12, column F). We then performed a BLAST search for the remaining 301 miRNA genes against the cat genome using human mature and precursor sequences as probes. We obtained a significant match for 164 distinct mature miRNAs encoded by 124 miRNA genes. However, only 33 out of the 124 miRNA genes exhibited conservation of their precursor sequences. Overall, our deep sequencing and *in-silico* analyses recovered 216 out of 268 conserved miRNA families (Supplementary Table S1.2, column BQ and S1.12, column C). A more thorough computational analysis would be necessary for a more accurate estimate. However, this goes beyond the purpose of our study.

### Tissue distribution

**Figure 2:**
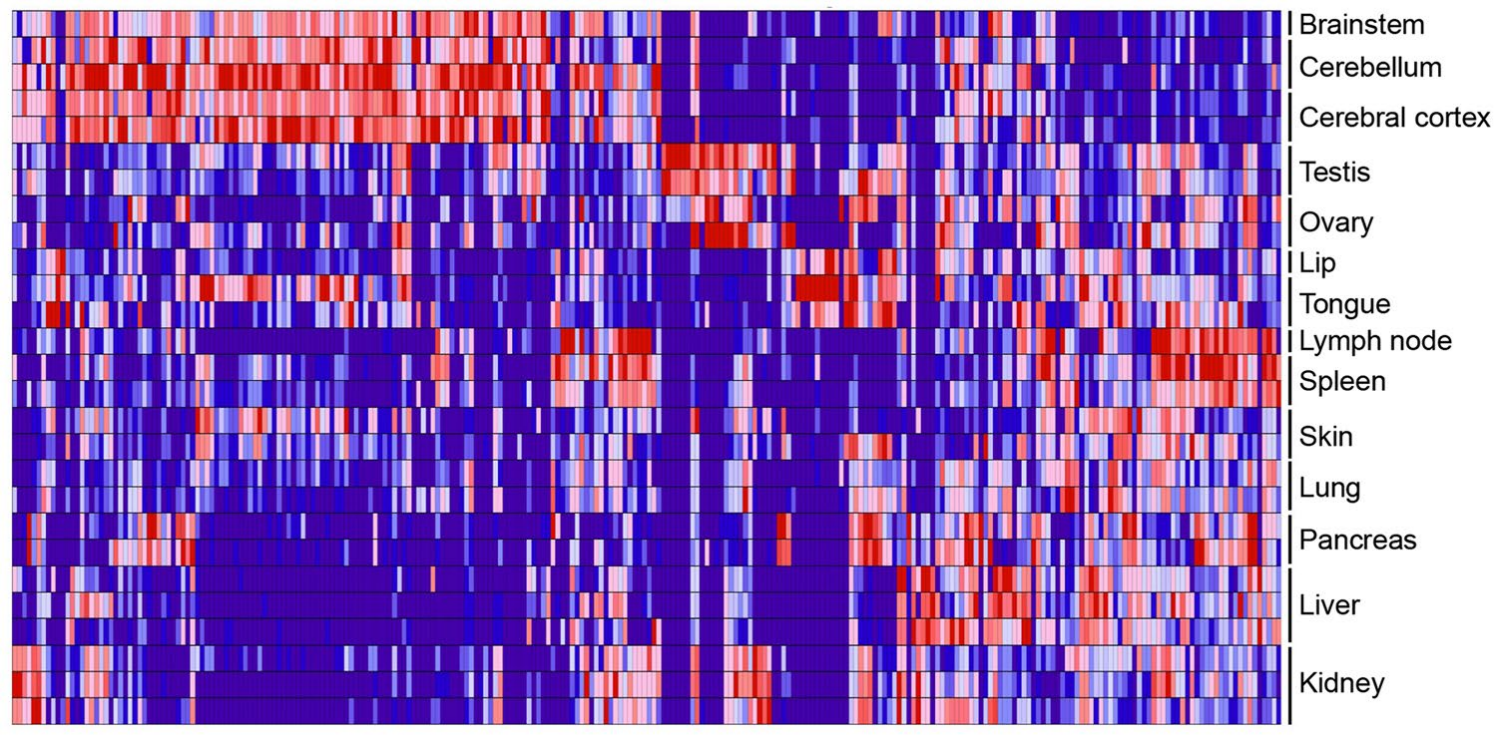
Heat map showing tissue-specific miRNA expression. Unsupervised hierarchical clustering was used to evaluate normalized expression of the most highly expressed mature miRNAs from each of the 271 precursors in all 27 samples. This analysis resulted in accurate clustering of all of the 27 samples within tissue types, as shown in the heat map.

Although most miRNAs were ubiquitously expressed, we observed significant differences in their distribution across the examined tissues. Figure S1 reports the number of different miRNA precursors and mature sequences across the analyzed samples. Unsupervised hierarchical clustering based on normalized expression of the most highly expressed mature miRNAs from each of the 271 precursors accurately distinguished between the different tissues (Fig. 2). We performed differential expression analysis of the mature miRNAs in order to identify signatures of tissue-specific miRNAs. We applied DESeq 2, a method for differential analysis of RNAseq count data, to perform pairwise comparisons of each tissue against all the others^16^. Uneven group sizes may result in lower power with the groups containing fewer samples. Nevertheless, our analysis identified groups of organ-enriched miRNAs in brain, liver, pancreas, lymph nodes, kidney, testis, spleen and lung (BH-adjusted p-value < 0.05). Table 1 summarizes all the significant tissue/organ-enriched and tissue/organ-specific miRNAs identified by our analysis (Details in Supplementary Table S1.2 and Supplementary Tables S3).

**Table 1:** Tissue enrichment of mature miRNA (Up-regulated miRNAs in each tissue/organ, BH-adjusted Pvalue < 0.05).

Tissue	Enriched miRNAs
Brain	fca-miR-124-3p, fca-miR-219-3p, fca-miR-124-5p, fca-miR-132-5p, fca-miR-219-5p, fca-miR-132-3p, fca-miR-138-2-3p, fca-miR-325-3p, fca-miR-433-3p, fca-miR-433-5p, fca-miR-323b-3p, fca-miR-chrE3_34323-5p, fca-miR-128-3p, fca-miR-325-5p, fca-miR-181c-5p, fca-miR-323a-3p, fca-miR-487a-5p, fca-miR-139-3p, fca-miR-432-3p, fca-miR-656-5p, fca-miR-1911-5p, fca-miR-410-3p, fca-miR-383-3p, fca-miR-105-5p, fca-miR-218-2-3p, fca-miR-487b-5p, fca-miR-1185-5p, fca-miR-149-5p, fca-miR-485-5p, fca-miR-chrE3_33972-3p, fca-miR-380-3p, fca-miR-656-3p, fca-miR-128-2-5p, fca-miR-chrD4_30107-3p, fca-miR-129-2-3p, fca-miR-chrA2_6163-3p, fca-miR-495-5p, fca-miR-129-5p, fca-miR-383-5p, fca-miR-485-3p, fca-miR-138-1-3p, fca-miR-7a-2-3p, fca-miR-543-3p, fca-miR-380-5p, fca-miR-382-3p, fca-miR-181d-5p, fca-miR-495-3p, fca-miR-329-5p, fca-miR-323a-5p, fca-miR-487a-3p, fca-miR-628-5p, fca-miR-382-5p, fca-miR-181c-3p, fca-miR-128-1-5p, fca-miR-129-1-3p, fca-miR-139-5p, fca-miR-370-5p, fca-miR-1251-3p, fca-miR-3085-3p, fca-miR-206-3p, fca-miR-1251-5p, fca-miR-889-3p, fca-miR-153-1-5p, fca-miR-218-5p, fca-miR-153-3p, fca-miR-543-5p, fca-miR-138-1-5p, fca-miR-889-5p, fca-miR-3085-5p, fca-miR-411-3p, fca-miR-chrA2_6163-5p, fca-miR-409-5p, fca-miR-138-2-5p, fca-miR-491-5p, fca-miR-491-3p, fca-miR-370-3p, fca-miR-432-5p, fca-miR-376a-5p, fca-miR-377-5p, fca-miR-335-5p, fca-miR-3958-3p, fca-miR-409-3p, fca-miR-758-5p, fca-miR-874-5p, fca-miR-758-3p, fca-miR-127-3p, fca-miR-134-3p, fca-miR-655-3p, fca-miR-885-5p, fca-miR-3959-3p, fca-miR-1296-5p, fca-miR-329-3p, fca-miR-7a-1-3p, fca-miR-340-3p, fca-miR-551b-3p, fca-miR-29b-2-5p, fca-miR-487b-3p, fca-miR-628-3p, fca-miR-1301-3p, fca-miR-340-5p, fca-miR-130b-5p, fca-miR-874-3p, fca-miR-181b-5p, fca-miR-103-5p, fca-miR-1343-3p, fca-miR-411-5p, fca-miR-299a-5p, fca-miR-379-5p, fca-let-7e-3p, fca-miR-664-3p, fca-miR-379-3p, fca-miR-98-3p, fca-miR-181a-1-3p, fca-miR-29b-3p, fca-miR-326-3p, fca-miR-3959-5p, fca-miR-chrE1_32174-3p, fca-miR-299a-1-5p, fca-miR-6529-3p, fca-miR-328-3p, fca-miR-chrB2_13690-3p, fca-miR-181a-5p, fca-miR-181b-1-3p, fca-miR-342-3p, fca-miR-374b-3p, fca-let-7e-5p, fca-miR-1249-3p, fca-miR-99b-3p, fca-miR-34a-3p, fca-miR-185-3p, fca-miR-134-5p, fca-miR-99b-5p, fca-miR-98-5p, fca-miR-125b-5p, fca-miR-185-5p, fca-miR-190a-3p, fca-miR-191-3p, fca-miR-29a-3p, fca-miR-29a-5p, fca-miR-6529-5p, fca-let-7g-3p, fca-miR-33-3p, fca-miR-331-3p, fca-let-7a-5p
Testis	fca-miR-chrX_38640-3p, fca-miR-514-5p, fca-miR-508-5p, fca-miR-8908n-5p, fca-miR-506-3p, fca-miR-508-3p, fca-miR-507a-3p, fca-miR-514-3p, fca-miR-8908n-3p, fca-miR-202-5p, fca-miR-302d-1-3p, fca-miR-chrX_38640-5p, fca-miR-chrX_38642-3p, fca-miR-202-3p, fca-miR-135b-5p
Oral	fca-miR-1-2-5p, fca-miR-chrA3_6354-5p, fca-miR-133a-3p, fca-miR-1-3p, fca-miR-1-1-5p, fca-miR-133a-5p, fca-miR-6715a-3p, fca-miR-296-5p, fca-miR-184-3p, fca-miR-296-3p, fca-miR-337-3p, fca-miR-23b-5p, fca-miR-22-5p, fca-miR-27b-5p, fca-miR-24-2-5p, fca-miR-24-3p
Liver	fca-miR-122-3p, fca-miR-3548-5p, fca-miR-3548-3p, fca-miR-483-3p, fca-miR-802-5p, fca-miR-192-5p, fca-miR-194-5p, fca-miR-192-3p, fca-miR-375-3p, fca-miR-148a-3p, fca-miR-148a-5p, fca-miR-101b-5p, fca-miR-193b-3p, fca-miR-193b-5p, fca-miR-193a-5p, fca-miR-365-3p, fca-miR-505-3p, fca-miR-9851-3p, fca-miR-374a-3p
Pancreas	fca-miR-375-3p, fca-miR-216a-5p, fca-miR-148a-3p, fca-miR-148a-5p, fca-miR-7-5p, fca-miR-215-5p, fca-miR-202-5p, fca-miR-802-5p, fca-miR-216a-3p, fca-miR-92a-3p, fca-miR-375-5p, fca-miR-802-3p, fca-miR-30b-5p, fca-miR-582-5p
Kidney	fca-miR-chrE3_33626-5p, fca-miR-196a-5p, fca-miR-chrE3_33626-3p, fca-miR-196b-5p, fca-miR-194-5p, fca-miR-204-5p, fca-miR-chrE2_33458-3p, fca-miR-30c-5p
Lymph node	fca-miR-150-5p
Spleen	fca-miR-144-3p, fca-miR-150-5p, fca-miR-486-5p, fca-miR-144-5p, fca-miR-18a-5p, fca-miR-chrE3_34145-5p, fca-miR-18b-5p, fca-miR-106a-5p, fca-miR-93-5p, fca-miR-106b-5p, fca-miR-106b-3p, fca-miR-93-3p, fca-miR-191-5p
Ovary	fca-miR-449-3p, fca-miR-449-5p, fca-miR-202-5p, fca-miR-34c-5p, fca-miR-34c-3p, fca-miR-506-3p, fca-miR-8908n-5p, fca-miR-508-3p, fca-miR-503-3p, fca-miR-514-3p, fca-miR-chrX_38640-3p, fca-miR-424-3p, fca-miR-514-5p
Lung	fca-miR-126-3p

### Arm selection preference

A miRNA precursor may encode one or two functional mature miRNAs, one from each arm of the hairpin (5p and 3p) and the most expressed product is commonly referred to as the dominant product^17^. Notably, our data showed that 201 out of the 271 identified precursors (74%), expressed both the 5p and 3p mature products in at least one tissue, and that 5p and 3p were the dominant forms for 53% and 47% of the precursors, respectively. Of the 70 remaining precursors, 27 expressed the 5p sequence only, while 43 expressed the 3p sequence only. We found that, based on the total number of reads per tissue, the choice of the dominant arm was consistent across all tissues for 252 out of 271 precursors (93%), while 19 of them exhibited arm switches (Supplementary Table S1.6)^18^.

Another common feature of miRNAs, which is widespread across different species, is that a single mature miRNA can be encoded by multiple precursors. Our analysis found 17 pairs and 3 triples of precursors encoding the same or highly similar mature products. For example, we found two precursors encoding let-7a-5p/let-7a-3p and three precursors encoding miR-199b-5p/miR-199b-3p. We also found a triple of precursors encoding the same dominant product, miR-7–5p, and slightly different versions of the non-dominant sequences: miR-7–1–3p, miR-7–2–3p and miR-7-3-3p (See Supplementary Table S1.2).

### Genomic Distribution and miRNA clusters

The miRNA precursors identified by our analysis were widely distributed throughout the genome with the exception of chromosome Y where no miRNAs were located. In humans, only two miRNAs have been found on chromosome Y. We discovered an average of 14.4 miRNAs per chromosome. Chromosomes B3 and B2 had the highest and the lowest number of miRNAs (if chromosome Y is excluded), 46 and 3, respectively. One miRNA was also assigned to the “unknown chromosome” chrUn_JH409706, and four were assigned to the fragment chrC1_JH408690_random. (See Supplementary Table S1.4).

Our analysis also identified 31 different miRNA clusters. A cluster is a group of precursors with an inter-miRNA gene distance of less than 10 kb on the same genomic strand^19^. Clustered miRNAs are generally transcribed as polycistronic primary transcripts (pri-miRNAs) and then processed into shorter pre-miRNAs to generate distinct mature miRNAs. It has been demonstrated that clustered miRNAs may regulate gene expression either individually or in combination in a coordinated manner and thus they may be functionally related.

**Figure 3:**
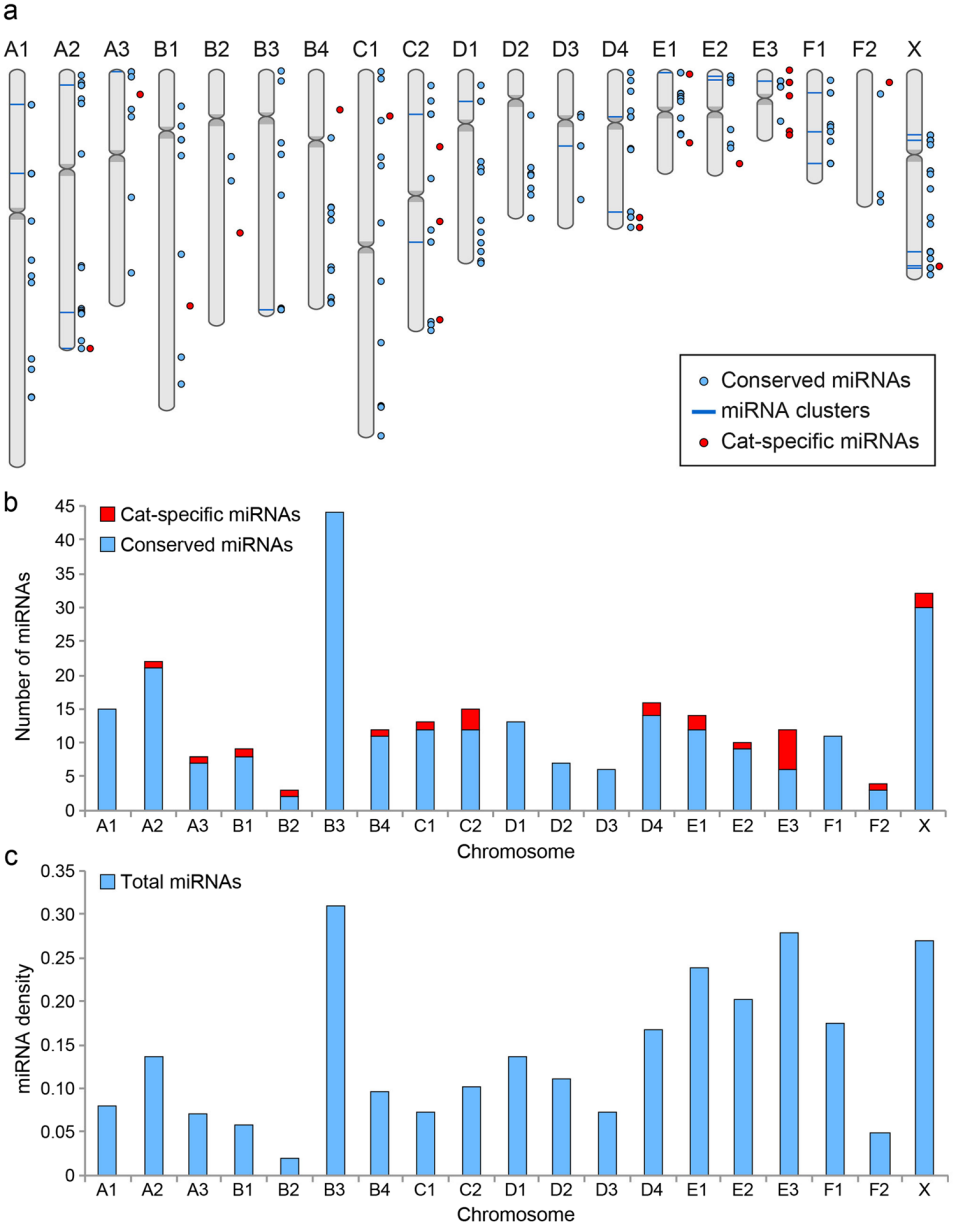
Genomic distribution of the identified miRNAs. (**a**) The figure shows the chromosomal distribution of cat-specific (red) and conserved (light blue) miRNAs. Clusters are represented by light blue bars. (**b**) The stacked column chart shows the total number of miRNAs per chromosome, highlighting the fractions of conserved (light blue) and novel, cat-specific (red) ones. Chromosomes A1, B3, D1, D2, D3 and F1 contain no cat-specific miRNAs, while Chromosome E3 has the highest number and percentage of cat-specific miRNAs (6 and 50%, respectively). (**c**) The chart shows the miRNA density per chromosome, calculated as the number of miRNAs per Mbp (Megabase pair).

Figure 3 depicts the genomic distribution of the miRNA genes and highlights their organization in clusters. The largest cluster was identified on chromosome B3 and consisted of 29 distinct miRNA genes. We observed a large area of about 10 Mb on chromosome X encompassing 5 close clusters containing a total of 16 different miRNAs. The size of the other clusters ranged from 2 to 5 miRNAs. Table 2 and Supplementary Table S1.5 provide a detailed report of the clusters, including conservation in human and dog.

**Table 2:** miRNA clusters identified by our analysis.

ID	miRNA gene	Chromosome	Conservation
1	fca-mir-15a, fca-mir-16-2	chrA1	hsa: chr13 ~, cfa: chr22 ~
2	fca-mir-17, fca-mir-18a, fca-mir-20a, fca-mir-20a, fca-mir-19b, fca-mir-92a	chrA1	hsa: chr13, cfa: chr22
3	fca-mir-302d-1, fca-mir-295, fca-mir-371	chrE2	—
4	fca-let-7e, fca-mir-99b	chrE2	hsa: chr19, cfa: chr1
5	fca-mir-24-2, fca-mir-27a	chrA2	hsa: chr19 ~, cfa: chr20 ~
6	fca-mir-181c, fca-mir-181d	chrA2	hsa: chr19, cfa: chr20
7	fca-mir-182, fca-mir-96, fca-mir-183	chrA2	hsa: chr7, cfa: chr14
8	fca-mir-153-2, fca-mir-chrA2_6163	chrA2	—
9	fca-mir-337, fca-mir-433, fca-mir-127, fca-mir-432	chrB3	hsa: chr14
10	fca-mir-379, fca-mir-411	chrB3	hsa: chr14, cfa: chr8
11	fca-mir-299a, fca-mir-380, fca-mir-323a, fca-mir-758, fca-mir-329, fca-mir-543, fca-mir-495, fca-mir-3958, fca-mir-376a-3, fca-mir-376c, fca-mir-376a-2, fca-mir-654, fca-mir-376ac, fca-mir-376a-1, fca-mir-1185, fca-mir-381, fca-mir-487b, fca-mir-889, fca-mir-655, fca-mir-3959, fca-mir-487a, fca-mir-382, fca-mir-134, fca-mir-485, fca-mir-323b, fca-mir-377, fca-mir-409, fca-mir-410, fca-mir-656, fca-mir-758, fca-mir-329, fca-mir-543, fca-mir-495, fca-mir-3958, fca-mir-376a-3, fca-mir-376c, fca-mir-376a-2, fca-mir-654, fca-mir-376ac, fca-mir-376a-1, fca-mir-1185, fca-mir-381, fca-mir-487b, fca-mir-889, fca-mir-655, fca-mir-3959, fca-mir-487a, fca-mir-382, fca-mir-134, fca-mir-485, fca-mir-323b, fca-mir-377, fca-mir-409, fca-mir-410, fca-mir-656	chrB3	hsa: chr14 ~, cfa: chr8 ~
12	fca-mir-221, fca-mir-222	chrX	hsa: chrX, cfa: chrX
13	fca-mir-532, fca-mir-188, fca-mir-362, fca-mir-660, fca-mir-502	chrX	hsa: chrX, cfa: chrX
14	fca-mir-363, fca-mir-20b, fca-mir-18b, fca-mir-106a	chrX	hsa: chrX, cfa: chrX
15	fca-mir-450b, fca-mir-542, fca-mir-503, fca-mir-424	chrX	hsa: chrX, cfa: chrX
16	fca-mir-chrX_38640, fca-mir-chrX_38642	chrX	—
17	fca-mir-506, fca-mir-507a	chrX	hsa: chrX ~, cfa: chrX
18	fca-mir-508, fca-mir-507b, fca-mir-514, fca-mir-8908n	chrX	hsa: chrX ~, cfa: chrX ~
19	fca-mir-195, fca-mir-497	chrE1	hsa: chr17, cfa: chr5
20	fca-let-7a-1, fca-mir-100	chrD1	hsa: chr11 ~
21	fca-mir-214, fca-mir-199a-1	chrF1	hsa: chr1 ~, cfa: chr7 ~
22	fca-mir-181b-1, fca-mir-181a-1	chrF1	hsa: chr9, cfa: chr9
23	fca-mir-215, fca-mir-194	chrF1	hsa: chr1 ~, cfa: chr38
24	fca-mir-133a-1, fca-mir-chrA3_7330, fca-mir-1-1	chrA3	hsa: chr18 ~, cfa: chr7 ~
25	fca-mir-133a-2, fca-mir-1-2	chrD3	hsa: chr18 ~
26	fca-mir-25, fca-mir-93, fca-mir-106b	chrE3	hsa: chr7, cfa: chr6
27	fca-mir-23b, fca-mir-27b, fca-mir-24-1	chrD4	hsa: chr9, cfa: chr1
28	fca-mir-181a-2, fca-mir-181b-2	chrD4	hsa: chr1, cfa: chr7
29	fca-mir-200a, fca-mir-429	chrC1_JH408690_random	hsa: chr1, cfa: chr5
30	fca-let-7c-2, fca-mir-99a	chrC2	hsa: chr21 ~, cfa: chr31 ~
31	fca-mir-16-1, fca-mir-15b	chrC2	hsa: chr13 ~, cfa: chr22 ~

Of the 31 clusters, 28 and 27 were completely or partially conserved in human and dog, respectively. Our analysis also revealed a cluster conserved in horse on chromosome X, consisting of miR-514 and miR-8908n. Among the non-conserved clusters, we identified a cluster with two cat-specific miRNAs on chromosome X (mir-chrX_38640 and mir-chrX_38642), a cluster containing miR-153 and a cat-specific miRNA on chromosome A2, and a cluster on chromosome E2 containing mir-295, mir-302d-1 and mir-371. miR-295 has not been characterized either in human or in dog, but orthologs were reported for mouse and rat, while miR-371 was conserved in many species, including human and dog, though clustered with other miRNAs. On the other hand, miR-302d-1 was a non-conserved precursor expressing a mature sequence similar to that of human miR-302d-3p.

### IsomiRs

The term isomiR refers to variations in size and sequence from the canonical reference miRNA sequence annotated in miRBase. Initially considered to be sequencing artifacts, isomiRs were recently reported as functional variants with a specific biological role, probably generated by variation in processing by Drosha and/or Dicer^20^. The majority of miRNA genes encode mature isomiRs and it appears that in some cases human miRNA genes express isomiRs as the dominant transcript in specific cell types. IsomiRs differ from canonical miRNAs by the addition or the removal of one or more bases at the 5′ and/or 3′ end of the sequence^21^. The additional bases usually match the reference precursor sequence, thus these isomiRs are called templated. Non-templated and polymorphic isomiRs are rarer isoforms which harbor nucleotide changes from the precursors at either the 5′/3′ ends or internally. These variants are probably the result of post-transcriptional modifications, i.e. editing. A-to-I editing is the most common form of RNA editing^22^.

**Figure 4:**
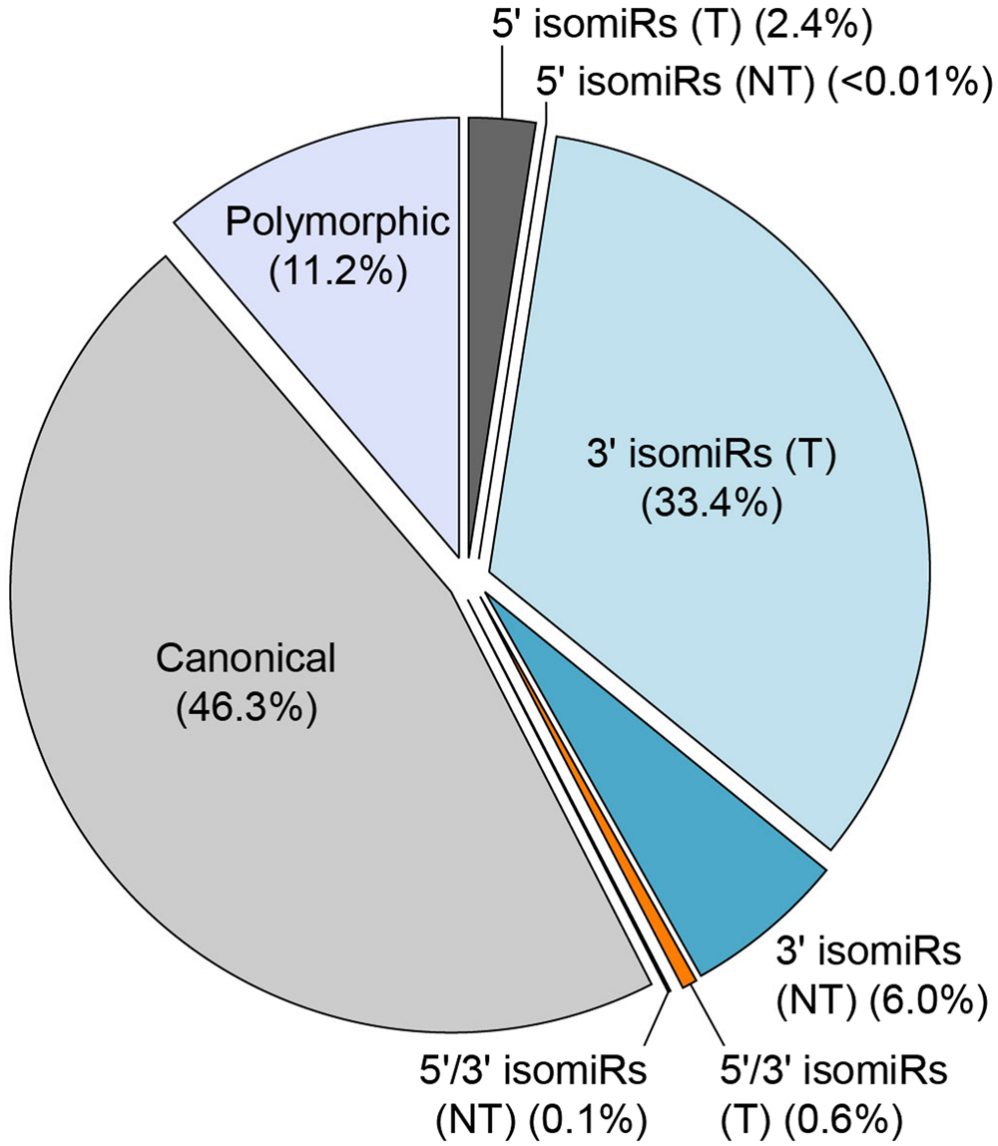
IsomiR distribution. The figure shows the distribution of the different types of isomiRs calculated on the total of mapped reads from all samples. The canonical form was predominant (46.3%), while the most frequent isomiR types were the templated (T) and non-templated (NT) 3′ isomiRs and the polymorphic isomiRs.

Our analysis classified the detected mature miRNA sequences into 5′/3′ templated and non-templated isomiRs, polymorphic isomiRs and canonical forms. Figure 4 shows the distribution of the different types of variants overall and in the different tissues. Overall, the most common products observed were the canonical form (46.3%), and the 3′ templated isoforms (33.4%), accounting for roughly 80% of the total number of reads. This was expected and in line with previous findings in other animal models, as 3′ isomiRs are much more likely to preserve the function of the canonical form. Target recognition, indeed, is mostly mediated by the seed sequence at the 5′ end of the miRNA, thus length modifications at the 3′ end are likely to not have any functional impact. The third most frequently observed type of variant was the polymorphic isomiR (11.2%), followed by 3′ non-templated and 5′ templated isomiRs (6.0% and 2.4%, respectively). The latter is particularly relevant, as 5′ modifications of the miRNA sequence may significantly affect target recognition and, consequently, be functionally important, as reported by recent work^23^. Non-templated 5′ isomiRs and simultaneous 5′/3′ templated and non-templated variants all together accounted for less than 1% of the total reads. Details on isomiRs are given in Supplementary Table S1.7.

### Validation of candidate miRNAs by Stem-Loop qRT-PCR

**Figure 5:**
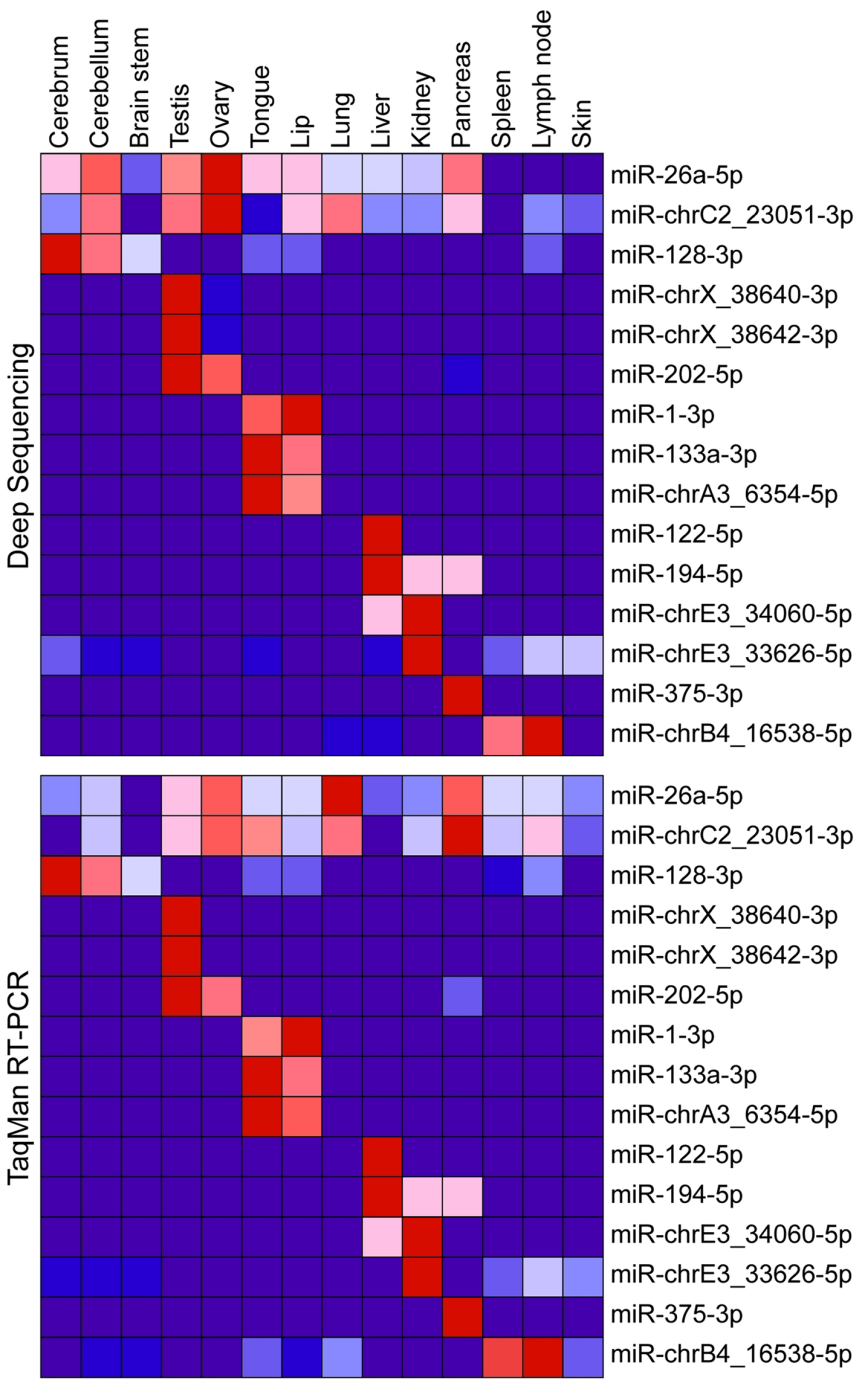
MiRNA validation. Nineteen miRNAs from our deep sequencing data were selected for further validation using real-time TaqMan® MicroRNA Assays, including nine novel, cat-specific miRNAs. miR-151 and miR-361 were expressed at very consistent levels in all of the deep sequencing samples (not shown) and, thus, qRT-PCR values were normalized to these two miRs. Figure shows heat maps of averaged and normalized miR counts from the deep sequencing data and of the normalized relative expression from qRT-PCR. All of the normalized deep sequencing and qRT-PCR values for each individual miR in each individual sample are given in Supplementary Table S1.10.

We confirmed the expression of 19 different microRNAs from our high-throughput sequencing data, including nine novel, cat-specific miRNAs. We chose representative miRNAs that were enriched in different tissues/organs to confirm that our high throughput sequencing data was quantitative (see Fig. 5). We also confirmed by qRT-PCR that fca-miR-chrD4_30107-3p and fca-miR-chrE3_34323-5p are enriched in the brain (data not shown). Each of these miRNAs was significantly enriched in specific tissues/organs, except for fca-miR-26a, fca-miR-151-3p, fca-miR-361-5p and fca-miR-chrC2_23051-3p, which were constitutively expressed in all tissues/organs examined. Heat maps of both the high-throughput sequencing and the stem-loop qRT-PCR data were virtually superimposable (Fig. 5 and Supplementary Table S1.10). Furthermore, a Pearson’s correlation coefficient analysis between the deep-sequencing and TaqMan data revealed that most miRNAs had a very high correlation between the two assays (see Supplementary Table S1.11). Only miR-26a-5p and miR-23051-3p were not significant, probably due to the fact that they were constitutively expressed. However, the correlation was still high (0.46 and 0.55).

### Comparison with previous studies

A recent study by Sun *et al*. performed high throughput sequencing on a feline kidney cell line (F81) before and after infection with mink enteritis virus (MEV)^13^. A careful comparison between our study and the Sun study revealed 186 common miRNAs (Supplementary Table S1.8). In addition, there were 264 unique miRNAs in our study and, surprisingly, 78 unique miRNAs in the Sun study.

In another study by Tamazian *et al*., a total of 370 unique feline miRNA genes were detected and mapped based upon homology to miRNA sequences from 36 species described in the miRBase database^10^. We have done a careful comparison between the lists of miRNAs generated in Tamazian’s and in our study (see Supplementary Table S1.9). We identified 213 miRNAs that had the same name and location between the two studies, confirming our assignment of these 213 miRNAs. In addition, there were 58 and 157 miRNAs that were unique to our study and Tamazian *et al*., respectively.

### Functional analysis of cat-specific miRNAs

Our analysis identified 25 novel, cat-specific miRNA precursors encoding 33 mature miRNAs. With the exception of seven miRNAs that were expressed in at least two different tissues, most novel miRNAs appeared to be tissue specific. In order to investigate the potential roles for the novel cat-specific miRNA candidates, we performed functional enrichment analysis of their predicted targets. In particular, we focused on 21 tissue-specific miRNAs expressed in brain, kidney, testis, spleen and ovary (see Table 1). Below, we briefly report a summary of the analysis. The detailed lists of targets and the complete results of the enrichment analysis are given as supplementary information (Tables S2.1-S2.5).

The analysis of the seven miRNAs expressed in the brain tissues analyzed (i.e. brain stem, cerebellum and cerebral cortex) revealed significant enrichment for pathways that are specific or relevant for brain functions or diseases, including *gap junction signaling*, *endothelin-1 signaling*, *ERK/MAPK signaling*, *GNRH signaling*, *p70S6K Signaling, CREB Signaling in Neurons*, *synaptic long term potentiation*, *glioma signaling* (BH-adjusted P < 0.05).

Similarly, the results for the three kidney-specific miRNAs, including fca- chrE3_33626-5p, which was validated by RT-PCR, showed significant enrichment for pathways that are relevant for kidney function, such as *AMPK signaling*, *cell cycle checkpoint control*, *Estrogen receptor signaling, Renin-Angiotensin signaling* and *Renal cell carcinoma signaling* (BH-adjusted P < 0.05).

The three miRNAs specifically expressed in testis, including fca-chrX-38640 and fca-chrX-38642, which were validated by RT-PCR, were significantly associated with pathways relevant for testis physiology, development and pathology, such as *IGF-1 signaling*, *PI3K/AKT signaling*, *Relaxin signaling*, *α-Adrenergic Signaling* and *TGF-β Signaling* (BH-adjusted P < 0.05).

The results for the spleen-specific miRNAs showed significant enrichment for pathways relevant for spleen function, such as *EGF Signaling*, *FLT3 Signaling in Hematopoietic Progenitor Cells*, *IGF-1 Signaling* and *CD40 Signaling* (BH-adjusted P < 0.05).

## Discussion

The domestic cat is not only a wonderful companion animal, but it also serves as a translational model for many human genetic, metabolic, and degenerative diseases and forms of cancer. In order to improve feline medicine and translate findings to humans, it is necessary that we have knowledge of the expression and regulation of miRNAs in normal feline tissues, since miRNAs play a crucial role in many biological processes and pathways and are implicated in most if not all diseases. In this study, we performed a genome-wide high-throughput sequencing analysis to define and characterize the feline miRNAome, which consists of 271 candidate feline miRNA precursors, encoding a total of 475 mature sequences, some of which appear to be tissue/organ-enriched and tissue/organ-specific.

Several of these miRNAs have also been shown to be tissue-enriched in other species, thus supporting the results of our analysis. For example, miR-1, miR-206 and miR-133a are highly enriched in cardiac and skeletal muscle and are collectively known as myomiRs^24^. They play a fundamental role in the regulation of muscle cell differentiation, development and maintenance^25^. Our analysis confirmed that they are also highly enriched in cat muscle tissue (lip and tongue samples). Other oral tissue-enriched miRNAs that we identified included miR-24, which has been reported as up-regulated in oral squamous cell carcinoma^26^, and miR-184, which was shown to be associated with anti-apoptotic and proliferative processes in tongue carcinoma^27^.

Our analysis identified 88 miRNAs specifically enriched in brain tissue. Among them, miR-219, miR-124, miR-153, miR-128, miR-132 and miR-139 are known to be brain enriched or brain-specific in other species and to be involved in several brain-specific functions^28^. For example, miR-219 has been reported to inhibit the proliferation, anchorage-independent growth and migration of glioma cells and to promote oligodendrocyte differentiation and myelination, while miR-124 has been shown to promote neurogenesis, inhibit proliferation of glioblastoma multiform cells and induce differentiation of brain tumor stem cells. Another important brain-enriched miRNA that was also found highly expressed in brain tissue by our analysis was miR-128. This miRNA has been reported to be involved in synaptogenesis, reduce neuroblastoma cell motility and invasiveness, and regulate apoptosis and inhibit proliferation and self-renewal in glioma.

Other tissue-specific miRNAs identified by our analysis that were also reported to be tissue-specific in other species include: miR-122, a liver-specific miRNA that functions as a tumor-suppressor gene in hepatocellular carcinoma^29^; miR-216, a pancreas-enriched miRNA, which has been reported as a marker for acute phase pancreatic injury and whose down-regulation is thought to be crucial in the development of pancreatic cancer^30^; miR-150, which our analysis reported as enriched in lymph nodes, is known to be expressed in mature B and T cells and, in particular, to regulate differentiation and the cytolytic effector function in CD8+ T cells^31^. Finally, our analysis identified a cluster of testis-enriched miRNAs located on chromosome X, including miR-506, miR-507, miR-508 and miR-514, that was previously reported as preferentially expressed in testis^32^.

Several studies have shown that arm selection preference may be tissue-specific^33^. We found that, based on the total number of reads per tissue, the choice of the dominant arm was consistent across all tissues for 252 out of 271 precursors (93%), while 19 of them exhibited arm switches. In some cases, arm switching appeared to be tissue specific, such as miR-378, whose dominant form was 5p in the ovary and 3p in all other tissues, and miR-493 and miR-140, whose dominant product was 5p in all tissues except skin, where the 3p form was expressed at a higher level. This is consistent with the fact that miR-140-3p was reported to be the dominant form in melanocytes and melanoma cells by previous studies^34^. In other cases, we observed less specific arm switching events across tissues. For example, the dominant product for let-7i was 5p in testis, ovary, lymph node, spleen, lung and oral tissue and 3p in brain, pancreas, skin, liver and kidney.

Arm switching can also be species specific^35^. For example, the dominant miR-10 sequences in fly (*Drosophila melanogaster*) and beetle (*Tribolium castaneum*) are processed from opposite arms^18^. Arm usage appears to be regulated through sequences in the primary miRNA sequence, outside the mature miRNA duplex, and the targets of miRNAs encoded from opposite arms may differ significantly. This strongly suggests that changes in arm preference throughout nature may have relevant functional consequences^36^.

Cluster analysis showed that, of the 31 clusters found in cat, 28 and 27 were completely or partially conserved in human and dog, respectively. For example, the miR-221/222 cluster on chromosome X was conserved in both human and dog also on chromosome X. These miRNAs, which also constitute a family, are known to be involved in cancer in humans^37^. They act either as oncogenes or tumor suppressors, depending on the type of tumor, and have been reported to have a role in drug resistance^38^. Recent studies, indeed, have revealed that targeting miR-221/222 may enhance sensitivity of cancer cell lines to drugs^39^. The miR-221/222 cluster is also considered a key player in vascular biology, as it contributes to vascular remodeling and plays a prominent role in atherosclerosis and in metabolic diseases, being involved in the regulation of insulin resistance^40^. Another important conserved cluster was the miR-17/92 cluster on chromosome A1. It is one of the most studied miRNA clusters and it is known to be involved in normal development and homeostasis, as well as in the pathogenesis of cancer, immune, cardiovascular and neurodegenerative diseases^41, 42^. Our analysis also revealed a cluster conserved in horse on chromosome X, consisting of miR-514 and miR-8908n. The human ortholog of miR-514 (miR-514a) was reported to be involved in cancer by initiating melanocyte transformation and promoting melanoma growth^43^, while miR-8908n was characterized in horse only and its function remains unknown.

A careful comparison between our study and a study on a feline kidney cell line^13^ revealed 186 common miRNAs. In addition, there were 264 unique miRNAs in our study and, surprisingly, 78 unique miRNAs in the Sun study. Our study had a broader scope and included 12 different tissues, thus the detection of a much larger number of miRNAs in our samples compared to just one cell line was reasonable to expect. However, it was surprising that Sun *et al*. identified 78 miRNAs that were not detected in our samples, especially since our study included three highly correlated kidney samples. Thus, these 78 miRNAs are probably induced during kidney cell immortalization/transformation and/or tissue culturing. Finally, there were 16 miRNAs that exhibited arm switches between the two studies. Five of these switches were very dramatic, going from > 90% of one arm in one study to > 90% of the opposite arm in the other study (fca-miR-1307, fca-miR-144, fca-miR-197, fca-miR-199 and fca-miR-33a). The most likely explanation for the differences observed between the two studies is that several miRNA genes are either turned off or on when cells are taken out of their primary environment and grown in tissue culture.

In another study, a total of 370 unique feline miRNA genes were detected and mapped based upon homology to miRNA sequences from 36 species described in the miRBase database^10^. We identified 213 miRNAs that were identical between the two studies, as well as 58 and 157 miRNAs that were unique to our study and Tamazian *et al*., respectively. The 157 miRNAs that we didn’t observe in our study probably include miRNAs that are not expressed in cat and/or are expressed in cat tissues/organs that we did not examine.

Our analysis also identified 25 novel, cat-specific miRNA precursors encoding 33 mature miRNAs, some of which were expressed in a tissue-specific manner and may be involved in the regulation of important biological processes and pathways. Functional analysis of their predicted targets confirmed significant enrichment for several pathways that are specific or relevant in the enriched tissues (Tables S2.1–S2.5), supporting our findings. However, further investigations will be necessary to confirm such roles and functions. Despite the remarkable advances made in recent years, computational prediction of miRNA targets still represents a challenge, as target recognition by miRNAs is a very complex and dynamic mechanism, still only partially understood^44^. Moreover, the UTR-ome of the cat has not yet been fully characterized, thus our analysis was limited to only ~8,300 UTR sequences, which is considerably less than the 20,285 putative genes present in cat^9^. Hence, the missing UTRs could affect the analysis. Nevertheless, this analysis provided useful information about the potential role of the candidate cat-specific miRNAs and support for their tissue specificity.

It has been argued that species-specific and lineage-specific microRNAs constitute a large proportion of miRNAs, and many of them have been recently identified through deep sequencing technologies in a variety of animals^45^. Although conserved miRNAs have been shown to be significantly more associated with disease than non-conserved miRNAs^46^, recent evidence suggests functional roles for species-specific miRNAs in a variety of biological processes, including apoptosis^47^, inflammation^48^ and nervous system development^49^. Silkworm-specific miRNAs were reported to have a functional preference for regulating genes involved in life-cycle-associated traits^50^. A more recent study has shown that a set of cattle-specific miRNAs may regulate the expression of target genes involved in insulin signaling through both conserved and cattle-specific binding sites, thus delineating gene expression divergence between ruminant and non-ruminant species^51^.

In conclusion, this study filled the gap in our knowledge regarding feline miRNAs by characterizing the feline miRNAome through deep sequencing of multiple cat tissues and organs. Our analysis identified 271 candidate feline miRNA precursors encoding a total of 475 mature sequences, including several novel cat-specific miRNAs. Further analyses were performed to characterize the discovered miRNAs in terms of genomic distribution, tissue expression, arm preference, isomiRs and function. Our comprehensive miRNA dataset provides the scientific community with a novel and useful resource for basic biology and veterinary clinical research, which may also inform and translate research findings for the benefit of humans.

## Materials and Methods

Tissues were obtained from control research cats and client-owned cats (with consent from the owners), collected at the time of euthanasia. The use of these research cats, as well as the client consent form, were approved by the Institutional Animal Care and Use Committee (IACUC) at The Ohio State University. All methods were performed in accordance with the IACUC guidelines and regulations.

### Tissue Harvesting and RNA isolation

The mirVana™ miRNA Isolation Kit (Life Technologies) was used to extract total RNA from lip (n = 1), brain (n = 5; brainstem, cerebellum and cerebral cortex), tongue (n = 2), testis (n = 2), ovary (n = 2), liver (n = 3), pancreas (n = 2), kidney (n = 3), lymph node, skin (n = 2), spleen (n = 2) and lung (n = 2). The tissues were obtained from various sources, including control research cats, client cats necropsied at the OSU Vet Med Center (with owner permission) and spayed and castrated cats from local shelters, as well as a few RNAs from Ziagen, Inc (Supplementary Table S1.1).

### Reverse transcription (RT) and SOLiD High-Throughput Sequencing

High-throughput sequencing of the purified cDNA libraries was performed using the NEBNext® Small RNA Library Prep Set for SOLiD™ (New England Biolabs), as follows: 1–5 μg of total RNA from each sample were ligated to 3′ and 5′ SR Adaptors provided in the kit. The adaptor-ligated RNAs were reverse transcribed and then amplified by PCR with primers specific to the adapter sequences. The reverse primers contained bar codes that allowed identification of individual samples. The barcodes were added to the 3′ end of the target sequence using a modified P2 adapter: SOLiD™ RNA Barcoding Kit, Module 1–16 (#4427046) and Module 17–32 (#4453189). SOLiD™ System barcoding enabled the assignment of a unique identifier to templated beads made from a single library. The PCR products were size-selected and purified on 8% acrylamide gels. Multiple batches of templated beads were pooled together for emulsion PCR. Templated sequencing beads were produced via emulsion PCR with Applied Biosystems’ EZ Bead system. The templated sequencing beads were sequenced on an Applied Biosystems SOLiD 5500 system, and the raw reads were pre-processed by the SOLiD software Lifescope. Processed high-quality sequences were analyzed by miRDeep2, a computational tool to map, analyze and score deep sequencing data for the identification of known and novel miRNAs^15^ (See “Bioinformatics and Statistical Analysis” for details). Sequencing data in fastq format is available at SRA (www.ncbi.nlm.nih.gov/sra) (Acc ID: SRP081245).

### Quantitative Stem-Loop Real-Time PCR

The expression of 19 different microRNAs from our high-throughput sequencing data was confirmed using real-time TaqMan^®^ MicroRNA Assays (LifeTechnologies): Briefly, 350 ng of each RNA sample were reverse transcribed with Multiscribe RT enzyme from the TaqMan® MicroRNA Reverse Transcription Kit (Life Technologies) and with pools containing mixtures of RT primers from the following TaqMan assays: mmu-miR-202 (assay no. 002579), hsa-miR-128a (no. 002216), hsa-miR-122a (no. 000445), hsa-miR-375 (no. 000564), hsa-miR-1 (no. 002222), hsa-miR-133a (no. 000458), hsa-miR-194 (no. 000493), hsa-miR-26a (no. 000405), mmu-miR-151 (no. 001190), hsa-miR-361 (no. 00054), as well as custom assays for fca-miR-chrX_38640-3p (no. CSAAYS8), fca-miR-chrC2_23051-3p (no. CT47VRZ), fca-miR-chrX_38642-3p (no. CTCE3VH), fca-miR-chrA3_6354-5p (no. CT2W7K6), fca-miR-chrE3_34060-5p (no. CT9HHWV), fca-miR-chrB4_16538-5p (no. CT32Z63), fca-miR-chrD4_30107-3p (no. CT7DPCX), fca-miR-chrE3_34323-5p (no. CTAAAAK) and fca-miR-chrE3_33626-5p (no. CS89JNF). The PCR reactions for each sample were performed in duplicate using TaqMan® Universal PCR Master Mix, no AmpErase® UNG (Life Technologies) and the reactions were run in a LightCycler480 (Roche) under the following PCR conditions: 10 min for 95 °C, and 40 cycles of 95 °C for 15 sec and 60 °C for 60 sec. All of the qRT-PCR data was normalized against the 2 miRNAs (miR-151 & 361) that had the lowest combined S.D. (S.D./Average normalized miR counts) in our deep-sequencing data across all 27 tissues, as recommended by Schwarzenbach *et al*.^52^. Finally, amplicons from each of the novel, cat-specific TaqMan miRNA assays were cloned and sequenced to verify that the proper miRNA was amplified with each TaqMan assay.

### Bioinformatics and Statistical Analysis

Raw reads obtained from the Applied Biosystems SOLiD 5500 system were pre-processed by the SOLiD software Lifescope, which filtered out low quality reads and performed mapping to the cat reference genome (*felCat5*, downloaded from UCSC: https://genome.ucsc.edu/cgi-bin/hgGateway?db=felCat5). High quality mapped reads were then processed by miRDeep2, a computational tool that identifies candidate miRNA precursors and their mature sequences from small RNA sequencing data. miRDeep2 makes use of other tools, such as the short read aligner Bowtie and the RNA secondary structure prediction tool RNAfold from the ViennaRNA package 2.0^53, 54^. Short reads were aligned to the reference genome, then all candidates whose structure and read signature were inconsistent with Drosha/Dicer processing were filtered out. Potential hairpin precursors were assigned a score according to a Bayesian probabilistic model of miRNA biogenesis.

No cat miRNAs were included in miRBase 21, thus all the detected miRNAs were considered novel. Candidate miRNAs detected by miRDeep2 were filtered by their scores. Then we applied filters based on score, expression and conservation by using our in-house developed ad-hoc scripts (Python and R) and a local version of BLAST. Similarly to what has been described in previous literature, we set a score cut-off threshold corresponding to the lowest score that yielded a signal-to-noise ratio higher than 10:1 (21.5:1)^55, 56^. Such value was 5, thus all the candidates with score below 5 were discarded. We kept all the candidate miRNAs expressed at detectable levels in at least one tissue, i.e. read count ≥10, this being a reasonable and commonly used count threshold^57^.

BLAST was used to filter out highly repetitive sequences and sequences matching other types of short RNA, and to evaluate conservation at the precursor and the mature sequence level.

Tissue distribution was evaluated by pairwise average-linkage unsupervised hierarchical clustering of the most highly expressed mature sequences from each precursor using Spearman’s rank correlation as a distance measure. Pairwise differential expression analysis was performed between different tissues by DESeq 2 in order to identify significant signatures of tissue-enriched miRNAs. Ad-hoc scripts were used to evaluate arm preference and detect arm switching across the samples, to evaluate the genomic distribution of the identified precursors and identify miRNA clusters, and to perform isomiRs analysis. Chromosome ideograms were created using the NCBI Genome Decoration Tool (http://www.ncbi.nlm.nih.gov/genome/tools/gdp).

We performed an *in-silico* analysis to investigate whether there was evidence in the cat genome of the conserved miRNAs that were missed by our analysis. We then performed a BLAST search for the remaining 301 miRNA genes against the cat genome using human mature and precursor sequences as probes. We discarded precursors exhibiting a match of less than 70% of their nucleotide bases and mature sequences without a complete seed match and EValue > 0.1.

miRNA Target prediction was performed by employing the tool miRiam and allowing canonical seeds only (6mer, 7mer-A1, 7mer-m8 and 8mer)^58^. The feline UTRome was retrieved from the UCSC Genome Browser (https://genome.ucsc.edu/cgi-bin/hgTables). Functional enrichment analysis of miRNA targets was performed using Ingenuity Pathway Analysis (IPA, http://www.ingenuity.com/products/ipa). Multiple testing correction was performed by using the Benjamini-Hochberg procedure.

The scripts used for the analysis are available upon request.

## Electronic supplementary material

Supplementary Figure 1

Supplementary Tables 1

Supplementary Tables 2

Supplementary Tables 3
