# Supplementary material for: Discovery and characterization of the feline miRNAome
Source: Sci Rep. 2017 Aug 23;7:9263. doi: 10.1038/s41598-017-10164-w (PMC5569061; doi:10.1038/s41598-017-10164-w)
Supplement: Supplementary file 1 — Supplementary Figure 1 [file 41598_2017_10164_MOESM1_ESM.pdf]

Discovery and characterization of the feline miRNAome

Alessandro Laganà, Wessel P. Dirksen, Wachiraphan Supsavhad, Ayse Selen Yilmaz, Hatice G. Ozer, James D. Feller, Kiersten A. Vala, Carlo M. Croce, and Thomas J. Rosol

SUPPLEMENTARY FIGURES

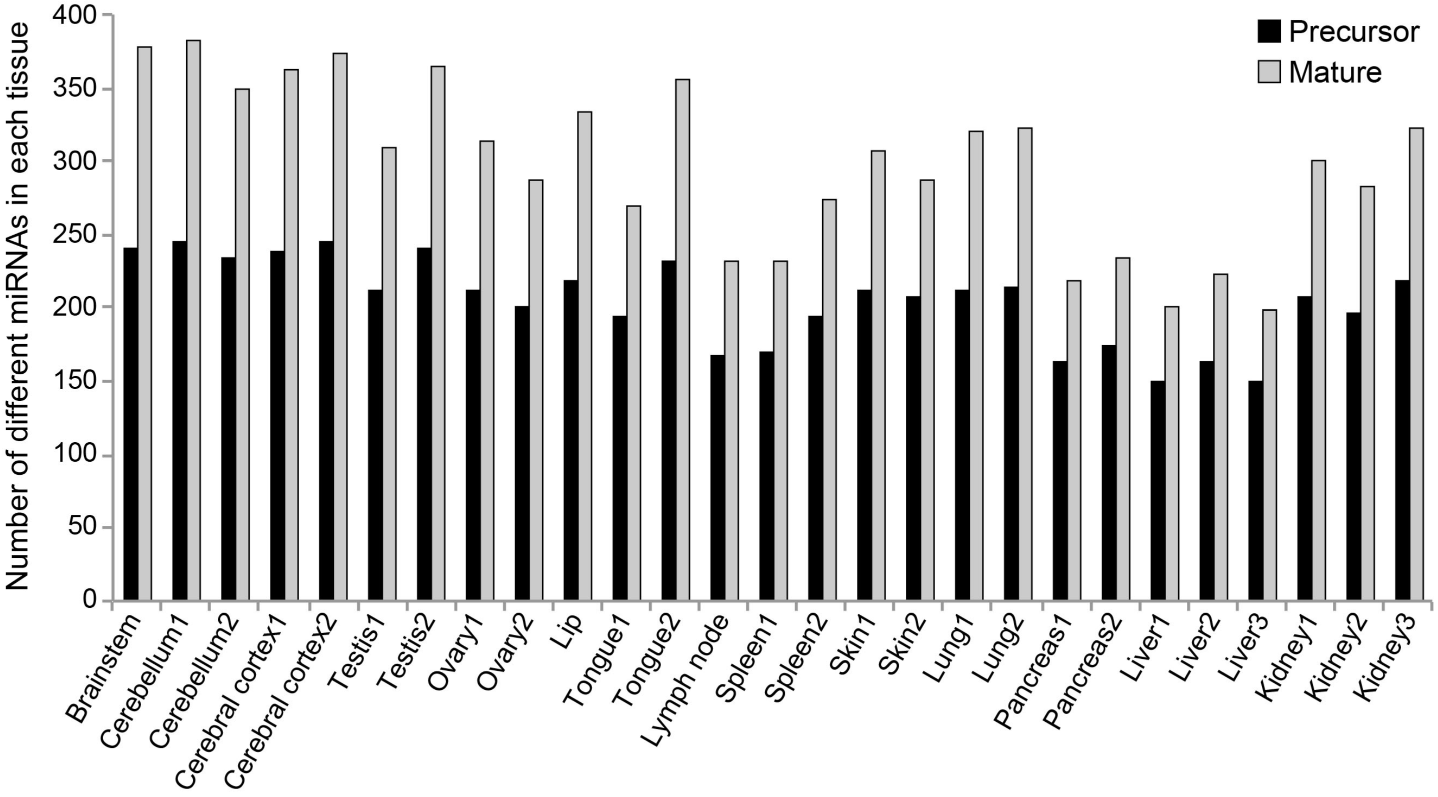

Fig. S1-Distribution of miRNA precursors and mature sequences across the analyzed samples. Graph shows total number of precursor and mature miRNAs in each tissue. Most of the precursors generate more than one mature miRNA. Thus, the number of mature miRNAs is greater than the number precursors.
